# *N*′-(4-Fluoro­benzyl­idene)-3,4,5-trimethoxy­benzohydrazide

**DOI:** 10.1107/S1600536808023702

**Published:** 2008-07-31

**Authors:** Dao-Hang He, Yong-Chuang Zhu, Zhuo-Ru Yang

**Affiliations:** a School of Chemistry and Chemical Engineering, South China University of Technology, Guangzhou 510640, People’s Republic of China

## Abstract

The title compound, C_17_H_17_FN_2_O_4_, is of inter­est due to its potential pharmaceutical and agrochemical activity. All three meth­oxy groups are twisted with respect to the attached aromatic ring [C—C—O—C torsion angles = 10.43 (18), 97.38 (14), −19.34 (17)°] and the phenyl ring makes a dihedral angle of 40.6 (2)° with the plane through the remaining atoms in the mol­ecule. Inter­molecular N—H⋯O hydrogen bonds link the mol­ecules into chains running along the *c* axis.

## Related literature

For related literature, see: Bernardino *et al.* (2006 ▶); Ganjali *et al.* (2006 ▶); Gardner *et al.* (1991 ▶); Lin *et al.* (2005 ▶); Patole *et al.* (2003 ▶); Liu *et al.* (2006 ▶); Zhou *et al.* (2005 ▶).
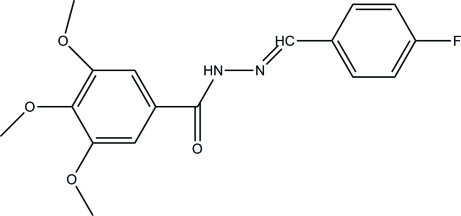


## Experimental

### 

#### Crystal data

C_17_H_17_FN_2_O_4_
*M* *_r_* = 332.33
Monoclinic, 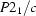

*a* = 7.9194 (4) Å
*b* = 26.2496 (13) Å
*c* = 8.1271 (4) Å
β = 105.5470 (10)°
*V* = 1627.65 (14) Å^3^
*Z* = 4
Mo *K*α radiation
μ = 0.10 mm^−1^
*T* = 173 (2) K
0.48 × 0.37 × 0.25 mm

#### Data collection

Bruker SMART 1000 CCD diffractometer
Absorption correction: multi-scan (*SADABS*; Sheldrick, 2003 ▶) *T*_min_ = 0.935, *T*_max_ = 0.974
9603 measured reflections
3558 independent reflections
2856 reflections with *I* > 2σ(*I*)
*R*_int_ = 0.018

#### Refinement

*R*[*F* ^2^ > 2σ(*F* ^2^)] = 0.037
*wR*(*F* ^2^) = 0.104
*S* = 1.07
3558 reflections
220 parameters
H-atom parameters constrained
Δρ_max_ = 0.27 e Å^−3^
Δρ_min_ = −0.20 e Å^−3^

### 

Data collection: *SMART* (Bruker, 2001 ▶); cell refinement: *SAINT-Plus* (Bruker, 2003 ▶); data reduction: *SAINT-Plus*; program(s) used to solve structure: *SHELXTL* (Sheldrick, 2008 ▶); program(s) used to refine structure: *SHELXTL*; molecular graphics: *SHELXTL*; software used to prepare material for publication: *SHELXTL*.

## Supplementary Material

Crystal structure: contains datablocks I, New_Global_Publ_Block. DOI: 10.1107/S1600536808023702/fl2210sup1.cif

Structure factors: contains datablocks I. DOI: 10.1107/S1600536808023702/fl2210Isup2.hkl

Additional supplementary materials:  crystallographic information; 3D view; checkCIF report

## Figures and Tables

**Table 1:** Hydrogen-bond geometry (Å, °)

*D*—H⋯*A*	*D*—H	H⋯*A*	*D*⋯*A*	*D*—H⋯*A*
N1—H1⋯O4^i^	0.88	1.96	2.8238 (13)	167

Symmetry code: (i) 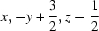.

**Fig. 1.**
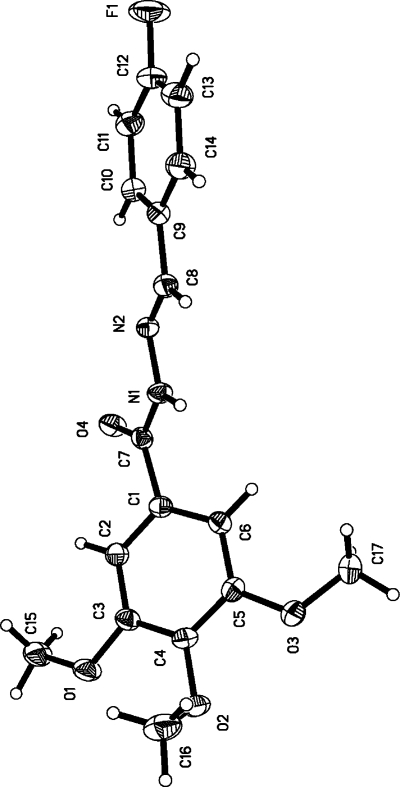
The Structure of the title compound, with the atom numbering. Displacement ellipsoids are the 50% probability level.

**Fig. 2.**
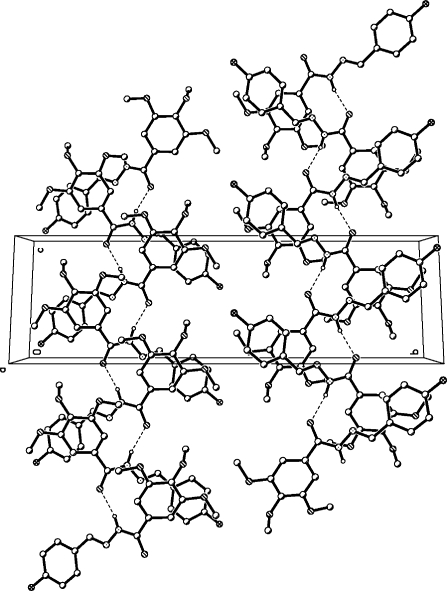
The packing of the title compound, viewed down the a axis. The dashed lines represent the hydrogen bonding interactions.

## supplementary crystallographic information

### Comment

Molecules involving Schiff bases are of interest due to their  biological activity as pharmaceuticals and agrochemicals (Bernardino *et al.*, 2006; Ganjali *et al.*, 2006; Gardner *et al.*, 1991; Patole *et al.*, 2003;). In addition, Schiff base ligands have attracted much attention because they can readily form stable complexes with most metal ions (Lin *et al.*, 2005; Zhou *et al.*, 2005). We report herein the synthesis and crystal structure of the Schiff base compound (I), obtained by the condensation of 3,4,5-trimethoxybenzohydrazide and 4-fluorobenzaldehyde.

All three methoxy groups are twisted with respect their attached aromatic ring (10.41°, 97.38°, -19.34°, respectively) and the phenyl ring itself makes a dihedral angle of 40.64° with the plane through the remaining atoms (C7 through F1) in the molecule(Fig. 1). Similar geometry has been observed in a related hydrazone analogue (Liu *et al.*, 2006). The bond lengths and bond angles are within normal ranges. Intermolecular N—H···O hydrogen bonds link the molecules into chains running along the *c* axis that help stabilize the molecular structure (Fig. 2).

### Experimental

A mixture of 3,4,5-trimethoxybenzohydrazide (1 mmol) and 4-fluorobenzaldehyde (1 mmol) in anhydrous ethanol (10 ml) was refluxed at 80 °C for 2 h. When the solution was cooled to room temperature(25 °C), some white needles separated out. After filtration, colorless single crystals suitable for X-ray analysis were obtained by slow evaporation of a methanol solution at room temperature.

### Refinement

All H atoms were placed in geometrically idealized positions and refined as riding, with N—H = 0.88 Å, C—H = 0.95 (aromatic and N=CH), 0.98 (methyl) Å and *U*_iso_(H) = *xU*_eq_(C, N), where *x* = 1.5 for the methyl, *x* = 1.2 for all other H atoms.

### Crystal data

**Table:**

C_17_H_17_FN_2_O_4_	*F*_000_ = 696
*M**_r_* = 332.33	*D*_x_ = 1.356 Mg m^−^^3^
Monoclinic, *P*2_1_/*c*	Mo *K*α radiation λ = 0.71073 Å
Hall symbol: -P 2ybc	Cell parameters from 5449 reflections
*a* = 7.9194 (4) Å	θ = 2.7–27.0º
*b* = 26.2496 (13) Å	µ = 0.11 mm^−^^1^
*c* = 8.1271 (4) Å	*T* = 173 (2) K
β = 105.5470 (10)º	Block, colorless
*V* = 1627.65 (14) Å^3^	0.48 × 0.37 × 0.25 mm
*Z* = 4	

### Data collection

**Table:**

Bruker SMART 1000 CCD diffractometer	3558 independent reflections
Radiation source: fine-focus sealed tube	2856 reflections with *I* > 2σ(*I*)
Monochromator: graphite	*R*_int_ = 0.018
*T* = 173(2) K	θ_max_ = 27.1º
ω scans	θ_min_ = 2.7º
Absorption correction: multi-scan(SADABS; Sheldrick, 2003)	*h* = −10→6
*T*_min_ = 0.935, *T*_max_ = 0.974	*k* = −33→33
9603 measured reflections	*l* = −10→10

### Refinement

**Table:**

Refinement on *F*^2^	Secondary atom site location: difference Fourier map
Least-squares matrix: full	Hydrogen site location: inferred from neighbouring sites
*R*[*F*^2^ > 2σ(*F*^2^)] = 0.037	H-atom parameters constrained
*wR*(*F*^2^) = 0.104	*w* = 1/[σ^2^(*F*_o_^2^) + (0.0561*P*)^2^ + 0.3099*P*] where *P* = (*F*_o_^2^ + 2*F*_c_^2^)/3
*S* = 1.07	(Δ/σ)_max_ = 0.001
3558 reflections	Δρ_max_ = 0.27 e Å^−^^3^
220 parameters	Δρ_min_ = −0.20 e Å^−^^3^
Primary atom site location: structure-invariant direct methods	Extinction correction: none

### Special details

**Table:**

Geometry. All e.s.d.'s (except the e.s.d. in the dihedral angle between two l.s. planes) are estimated using the full covariance matrix. The cell e.s.d.'s are taken into account individually in the estimation of e.s.d.'s in distances, angles and torsion angles; correlations between e.s.d.'s in cell parameters are only used when they are defined by crystal symmetry. An approximate (isotropic) treatment of cell e.s.d.'s is used for estimating e.s.d.'s involving l.s. planes.
Refinement. Refinement of *F*^2^ against ALL reflections. The weighted *R*-factor *wR* and goodness of fit *S* are based on *F*^2^, conventional *R*-factors *R* are based on *F*, with *F* set to zero for negative *F*^2^. The threshold expression of *F*^2^ > σ(*F*^2^) is used only for calculating *R*-factors(gt) *etc*. and is not relevant to the choice of reflections for refinement. *R*-factors based on *F*^2^ are statistically about twice as large as those based on *F*, and *R*- factors based on ALL data will be even larger.

### Fractional atomic coordinates and isotropic or equivalent isotropic displacement parameters (Å^2^)

**Table:**

	*x*	*y*	*z*	*U*_iso_*/*U*_eq_	
C1	0.63386 (15)	0.68413 (4)	0.23670 (15)	0.0213 (3)	
C2	0.65648 (16)	0.63159 (5)	0.23753 (15)	0.0234 (3)	
H2	0.7222	0.6148	0.3378	0.028*	
C3	0.58205 (16)	0.60404 (5)	0.09044 (16)	0.0242 (3)	
C4	0.48089 (16)	0.62887 (5)	−0.05539 (15)	0.0240 (3)	
C5	0.44513 (15)	0.68070 (5)	−0.04828 (15)	0.0233 (3)	
C6	0.52479 (15)	0.70882 (4)	0.09643 (15)	0.0222 (3)	
H6	0.5051	0.7445	0.0997	0.027*	
C7	0.73648 (15)	0.71281 (4)	0.39003 (15)	0.0214 (2)	
C8	0.94162 (16)	0.83150 (5)	0.43795 (16)	0.0249 (3)	
H8	0.9218	0.8379	0.3192	0.030*	
C9	1.03374 (16)	0.86967 (5)	0.56060 (16)	0.0249 (3)	
C10	1.06439 (16)	0.86332 (5)	0.73717 (17)	0.0274 (3)	
H10	1.0287	0.8327	0.7804	0.033*	
C11	1.14578 (19)	0.90092 (5)	0.84950 (18)	0.0335 (3)	
H11	1.1672	0.8965	0.9694	0.040*	
C12	1.19497 (19)	0.94501 (5)	0.78240 (19)	0.0365 (3)	
C13	1.1702 (2)	0.95280 (5)	0.6106 (2)	0.0373 (3)	
H13	1.2076	0.9834	0.5690	0.045*	
C14	1.08883 (19)	0.91458 (5)	0.49972 (18)	0.0324 (3)	
H14	1.0704	0.9191	0.3802	0.039*	
C15	0.6792 (2)	0.52504 (5)	0.22518 (19)	0.0372 (3)	
H15A	0.8023	0.5352	0.2688	0.056*	
H15B	0.6727	0.4885	0.1999	0.056*	
H15C	0.6166	0.5324	0.3114	0.056*	
C16	0.5363 (2)	0.59696 (6)	−0.30234 (19)	0.0423 (4)	
H16A	0.5561	0.6304	−0.3474	0.063*	
H16B	0.4903	0.5734	−0.3974	0.063*	
H16C	0.6472	0.5838	−0.2300	0.063*	
C17	0.25329 (17)	0.74833 (5)	−0.17173 (17)	0.0300 (3)	
H17A	0.2088	0.7471	−0.0703	0.045*	
H17B	0.1561	0.7551	−0.2729	0.045*	
H17C	0.3406	0.7755	−0.1584	0.045*	
F1	1.27194 (14)	0.98260 (3)	0.89208 (12)	0.0556 (3)	
N1	0.79692 (13)	0.75849 (4)	0.35482 (12)	0.0224 (2)	
H1	0.7790	0.7684	0.2481	0.027*	
N2	0.88701 (13)	0.78964 (4)	0.48641 (13)	0.0225 (2)	
O1	0.60103 (13)	0.55283 (3)	0.07321 (12)	0.0323 (2)	
O2	0.41211 (12)	0.60182 (3)	−0.20300 (11)	0.0294 (2)	
O3	0.33239 (12)	0.70067 (3)	−0.19160 (11)	0.0308 (2)	
O4	0.76599 (13)	0.69488 (3)	0.53497 (11)	0.0292 (2)	

### Atomic displacement parameters (Å^2^)

**Table:**

	*U*^11^	*U*^22^	*U*^33^	*U*^12^	*U*^13^	*U*^23^
C1	0.0232 (6)	0.0219 (6)	0.0196 (6)	−0.0017 (5)	0.0073 (5)	−0.0015 (4)
C2	0.0266 (6)	0.0214 (6)	0.0214 (6)	−0.0002 (5)	0.0049 (5)	0.0018 (5)
C3	0.0279 (6)	0.0181 (6)	0.0265 (6)	−0.0025 (5)	0.0071 (5)	−0.0009 (5)
C4	0.0250 (6)	0.0233 (6)	0.0224 (6)	−0.0051 (5)	0.0040 (5)	−0.0036 (5)
C5	0.0218 (6)	0.0253 (6)	0.0216 (6)	−0.0008 (5)	0.0038 (5)	0.0020 (5)
C6	0.0243 (6)	0.0188 (6)	0.0236 (6)	0.0000 (5)	0.0067 (5)	0.0000 (5)
C7	0.0248 (6)	0.0208 (6)	0.0186 (6)	0.0033 (5)	0.0059 (5)	−0.0009 (4)
C8	0.0272 (6)	0.0243 (6)	0.0217 (6)	0.0010 (5)	0.0039 (5)	−0.0024 (5)
C9	0.0222 (6)	0.0220 (6)	0.0289 (7)	0.0012 (5)	0.0040 (5)	−0.0035 (5)
C10	0.0257 (6)	0.0251 (6)	0.0296 (7)	−0.0002 (5)	0.0044 (5)	−0.0019 (5)
C11	0.0352 (7)	0.0342 (7)	0.0276 (7)	−0.0028 (6)	0.0026 (6)	−0.0050 (6)
C12	0.0365 (8)	0.0271 (7)	0.0398 (8)	−0.0053 (6)	−0.0002 (6)	−0.0114 (6)
C13	0.0407 (8)	0.0254 (7)	0.0429 (8)	−0.0066 (6)	0.0061 (6)	0.0000 (6)
C14	0.0369 (7)	0.0286 (7)	0.0295 (7)	−0.0027 (6)	0.0054 (6)	0.0002 (5)
C15	0.0513 (9)	0.0191 (6)	0.0347 (8)	0.0005 (6)	0.0004 (6)	0.0027 (5)
C16	0.0440 (9)	0.0516 (9)	0.0317 (8)	−0.0087 (7)	0.0109 (7)	−0.0147 (7)
C17	0.0272 (7)	0.0311 (7)	0.0302 (7)	0.0052 (5)	0.0049 (5)	0.0054 (5)
F1	0.0731 (7)	0.0357 (5)	0.0470 (6)	−0.0186 (5)	−0.0030 (5)	−0.0153 (4)
N1	0.0281 (5)	0.0222 (5)	0.0156 (5)	−0.0024 (4)	0.0034 (4)	−0.0018 (4)
N2	0.0227 (5)	0.0221 (5)	0.0208 (5)	0.0009 (4)	0.0026 (4)	−0.0048 (4)
O1	0.0456 (6)	0.0178 (4)	0.0289 (5)	−0.0011 (4)	0.0021 (4)	−0.0013 (4)
O2	0.0314 (5)	0.0283 (5)	0.0252 (5)	−0.0056 (4)	0.0018 (4)	−0.0067 (4)
O3	0.0347 (5)	0.0280 (5)	0.0235 (5)	0.0041 (4)	−0.0028 (4)	0.0004 (4)
O4	0.0448 (6)	0.0223 (4)	0.0186 (4)	−0.0007 (4)	0.0055 (4)	0.0006 (3)

### Geometric parameters (Å, °)

**Table:**

C1—C2	1.3905 (17)	C11—C12	1.379 (2)
C1—C6	1.3921 (17)	C11—H11	0.9500
C1—C7	1.4957 (16)	C12—F1	1.3596 (15)
C2—C3	1.3862 (17)	C12—C13	1.372 (2)
C2—H2	0.9500	C13—C14	1.3866 (19)
C3—O1	1.3639 (14)	C13—H13	0.9500
C3—C4	1.4008 (17)	C14—H14	0.9500
C4—O2	1.3748 (14)	C15—O1	1.4252 (16)
C4—C5	1.3941 (17)	C15—H15A	0.9800
C5—O3	1.3679 (14)	C15—H15B	0.9800
C5—C6	1.3886 (16)	C15—H15C	0.9800
C6—H6	0.9500	C16—O2	1.4354 (17)
C7—O4	1.2312 (14)	C16—H16A	0.9800
C7—N1	1.3499 (15)	C16—H16B	0.9800
C8—N2	1.2808 (16)	C16—H16C	0.9800
C8—C9	1.4629 (17)	C17—O3	1.4276 (15)
C8—H8	0.9500	C17—H17A	0.9800
C9—C14	1.3931 (18)	C17—H17B	0.9800
C9—C10	1.3997 (18)	C17—H17C	0.9800
C10—C11	1.3812 (18)	N1—N2	1.3829 (13)
C10—H10	0.9500	N1—H1	0.8800
			
C2—C1—C6	121.11 (11)	F1—C12—C11	118.32 (13)
C2—C1—C7	117.04 (10)	C13—C12—C11	123.28 (13)
C6—C1—C7	121.82 (10)	C12—C13—C14	117.94 (13)
C3—C2—C1	119.26 (11)	C12—C13—H13	121.0
C3—C2—H2	120.4	C14—C13—H13	121.0
C1—C2—H2	120.4	C13—C14—C9	121.10 (13)
O1—C3—C2	124.72 (11)	C13—C14—H14	119.5
O1—C3—C4	115.18 (11)	C9—C14—H14	119.5
C2—C3—C4	120.10 (11)	O1—C15—H15A	109.5
O2—C4—C5	120.19 (11)	O1—C15—H15B	109.5
O2—C4—C3	120.06 (11)	H15A—C15—H15B	109.5
C5—C4—C3	119.70 (11)	O1—C15—H15C	109.5
O3—C5—C6	124.21 (11)	H15A—C15—H15C	109.5
O3—C5—C4	115.61 (10)	H15B—C15—H15C	109.5
C6—C5—C4	120.17 (11)	O2—C16—H16A	109.5
C5—C6—C1	119.17 (11)	O2—C16—H16B	109.5
C5—C6—H6	120.4	H16A—C16—H16B	109.5
C1—C6—H6	120.4	O2—C16—H16C	109.5
O4—C7—N1	123.72 (11)	H16A—C16—H16C	109.5
O4—C7—C1	121.75 (11)	H16B—C16—H16C	109.5
N1—C7—C1	114.51 (10)	O3—C17—H17A	109.5
N2—C8—C9	121.72 (11)	O3—C17—H17B	109.5
N2—C8—H8	119.1	H17A—C17—H17B	109.5
C9—C8—H8	119.1	O3—C17—H17C	109.5
C14—C9—C10	118.70 (12)	H17A—C17—H17C	109.5
C14—C9—C8	118.95 (12)	H17B—C17—H17C	109.5
C10—C9—C8	122.33 (12)	C7—N1—N2	120.01 (9)
C11—C10—C9	120.96 (13)	C7—N1—H1	120.0
C11—C10—H10	119.5	N2—N1—H1	120.0
C9—C10—H10	119.5	C8—N2—N1	114.60 (10)
C12—C11—C10	118.01 (13)	C3—O1—C15	116.73 (10)
C12—C11—H11	121.0	C4—O2—C16	111.45 (10)
C10—C11—H11	121.0	C5—O3—C17	116.46 (10)
F1—C12—C13	118.41 (13)		
			
C6—C1—C2—C3	6.01 (18)	N2—C8—C9—C10	−1.37 (19)
C7—C1—C2—C3	−171.90 (11)	C14—C9—C10—C11	0.73 (19)
C1—C2—C3—O1	177.13 (12)	C8—C9—C10—C11	−177.48 (12)
C1—C2—C3—C4	−1.92 (18)	C9—C10—C11—C12	0.4 (2)
O1—C3—C4—O2	−1.36 (17)	C10—C11—C12—F1	178.69 (12)
C2—C3—C4—O2	177.77 (11)	C10—C11—C12—C13	−1.3 (2)
O1—C3—C4—C5	176.35 (11)	F1—C12—C13—C14	−178.91 (13)
C2—C3—C4—C5	−4.53 (18)	C11—C12—C13—C14	1.1 (2)
O2—C4—C5—O3	3.48 (17)	C12—C13—C14—C9	0.1 (2)
C3—C4—C5—O3	−174.22 (11)	C10—C9—C14—C13	−1.0 (2)
O2—C4—C5—C6	−175.29 (11)	C8—C9—C14—C13	177.31 (12)
C3—C4—C5—C6	7.01 (18)	O4—C7—N1—N2	−4.82 (18)
O3—C5—C6—C1	178.34 (11)	C1—C7—N1—N2	177.12 (9)
C4—C5—C6—C1	−3.01 (17)	C9—C8—N2—N1	177.75 (10)
C2—C1—C6—C5	−3.55 (17)	C7—N1—N2—C8	178.47 (11)
C7—C1—C6—C5	174.26 (11)	C2—C3—O1—C15	10.43 (18)
C2—C1—C7—O4	−35.84 (16)	C4—C3—O1—C15	−170.49 (12)
C6—C1—C7—O4	146.27 (12)	C5—C4—O2—C16	97.38 (14)
C2—C1—C7—N1	142.27 (11)	C3—C4—O2—C16	−84.92 (15)
C6—C1—C7—N1	−35.63 (16)	C6—C5—O3—C17	−19.34 (17)
N2—C8—C9—C14	−179.58 (12)	C4—C5—O3—C17	161.96 (11)

### Hydrogen-bond geometry (Å, °)

**Table:**

*D*—H···*A*	*D*—H	H···*A*	*D*···*A*	*D*—H···*A*
N1—H1···O4^i^	0.88	1.96	2.8238 (13)	167

Symmetry codes: (i) *x*, −*y*+3/2, *z*−1/2.
